# The Middlesex Hospital

**Published:** 1892-12-03

**Authors:** 


### HOSPITAL MEETINGS.

### THE MIDDLESEX HOSPITAL.

The quarterly meeting of the governors was held at the Middlesex Hospital on November 24th, at noon, Mr. Custance being in the chair. Amongst those present at the meeting were: Mr. Bell Sedgwick, Colonel Holloway, Mr. T. B. Bancroft, Mr. Jarrett, Mr. G. B. Hudson, M.P., Mr. Kegan Paul, Mr. Thurlow, Mr. J. Larkins, J.P., and Mr. Brindley.

It was decided to present a gratuity of £300 to the Chaplain (Mr. Brindley), who has resigned his appointment after fourteen years' service in the hospital. The posts of warden of the Medical College and of the chaplaincy are combined at the Middlesex Hospital. Mr. Brindley has resigned, not desiring to hold both offices, which the committee do not consider it advisable to separate.

Dr. Edwards Stuart was unanimously elected as a governor to fill the vacancy caused by Mr. Attenborough's lamented decease.

It having become possible to carry out the very desirable arrangement of having resident ward-maids, accommodation has been provided in a house in Cleveland Street, which will be connected with the hospital by a covered way.

Additional appliances for the extinction of fire have been recently placed in the Nurses' Home, Hospital, and Medical College, outside the Chemical Laboratory.

On receiving the report of the sums collected in boxes within and without the hospital, it was satisfactory to note an increase in each instance had taken place since last year, as follows :—

| | 1891. | 1892. |
|---|---|---|
| Outside boxes ......... | £198 11 6 | ...... £225 0 0 |
| Inside boxes............ | 36 18 0 | ...... 49 2 6 |
| Samaritan Fund ...... | 12 2 6 | ...... 18 1 0 |
| | £247 12 0 | £292 3 6 |

A legacy of £5,000 from the late James Stuart Forbes, Esq., was mentioned, and a proposal was carried to make his executors (Mr. Tweedie and Mr. Forbes Tweedie) life governors of the hospital.

Contributions were reported as having been received for necessary repairs to the hospital chapel, and various donations of fruit, game, vegetables, and a hundred flannel jackets were also gratefully mentioned.

